# Temperature-induced changes of spike timing precision and network synchronisation

**DOI:** 10.1186/1471-2202-16-S1-P219

**Published:** 2015-12-18

**Authors:** Jan-Hendrik Schleimer, Janina Hesse, Susanne Schreiber

**Affiliations:** 1 Institute for Theoretical Biology, Institute for Biology, Humboldt University, Berlin, Germany; 2 Bernstein Centre for Computational Neuroscience, Berlin, Germany

## 

Changes in the temperature of neuronal tissue influence electrical activity in non-trivial ways [1]. Despite a central regulation of body temperature in mammals, small changes in temperature can have substantial effects. For example, increases in brain temperature can in rare cases even induce epileptic seizures [2]. At the level of single cells, temperature affects ion channels: an increase in temperature predominantly speeds up their opening and closing rates and increases their maximal conductances. While the temperature dependence of nervous system function is widely acknowledged, we currently still lack a full understanding of the effects of temperature on local circuits.

Here, we investigate the impact of temperature on spike timing precision and network synchronisation. It is known that properties of individual ion channel types can modify spiking regularity of single neurons and network synchronisation. To study temperature-induced changes of these observables, multiple ion channels have to be considered simultaneously. Our approach is based on analytical simplification and numerical continuation. Spike jitter is analysed in temperature-dependent conductance-based models with ion channel stochasticity. Below threshold, the dynamics are appropriately described by a coloured noise escape problem, and, above threshold, by a phase-oscillator description. We show that for common, physiological parameter combinations, an increase in temperature reduces spike jitter. Below threshold, this can be explained by a temperature-induced redistribution of current noise power to higher frequencies, which are filtered out by the membrane impedance. Above threshold, the faster dynamics at elevated temperatures alter the ion channels' phase response curves and hence their phase susceptibility. These results are interpreted in the context of network synchronisation by showing the effect of temperature on the entrainment region of deterministic models.
